# Multiple mesenteric well-differentiated liposarcoma complicated by purulent inflammation: A case report

**DOI:** 10.3892/ol.2015.2847

**Published:** 2015-01-05

**Authors:** WEI GAO, HUAIZHOU WANG, JINYU LIU, FUJIANG WANG, JIANJUN DONG, JUNZU GENG

**Affiliations:** 1 Department of Radiology, Yantai Yuhuangding Hospital, Yantai, Shandong 264000, P.R. China; 2 Department of Surgery, Yantai Kouqiang Hospital, Yantai, Shandong 264000, P.R. China

**Keywords:** multiple, mesenteric, well-differentiated liposarcoma, computed tomography, purulent inflammation

## Abstract

Multiple mesenteric well-differentiated (WD) liposarcoma is an extremely rare entity. The present study describes a case of multiple mesenteric WD liposarcoma, complicated by purulent inflammation, in a 59-year-old male who presented with abdominal pain and pyrexia of unknown origin. A computed tomography scan of the abdomen revealed a large, non-encapsulated mass in the abdomino-pelvic cavity, which was characterized by two components, a main portion of fatty density and a non-adipose solid portion. A re-evaluated CT scan, performed eight days later, revealed an enlargement of the non-adipose mass. A laparotomy was performed, and numerous separated fatty nodules and masses of various sizes were identified within the mesentery of the small intestine. The histological findings were consistent with an adipocytic subtype of multiple mesenteric WD liposarcoma, with the largest of the tumors complicated by purulent inflammation. The multiplicity of these tumors and the concurrent purulent inflammation in the present case make it unique.

## Introduction

Well-differentiated (WD) liposarcoma is the most common subtype of liposarcoma, accounting for 40–45% of all cases (1). The most frequent site for WD liposarcoma is in the deep soft tissues of the thigh, followed by the retroperitoneum, paratesticular region and mediastinum (2). Other sites are uncommon and in particular, primary WD liposarcoma of the mesentery is rare. Multiple mesenteric WD liposarcoma is extremely rare, with only one reported case in the English literature (2). The present study describes a case of multiple mesenteric WD liposarcoma, which was complicated by purulent inflammation and difficult to diagnose pre-operatively, and discusses the radiological findings. Informed consent for the study was obtained from the patient’s family.

## Case report

**Figure 1:**
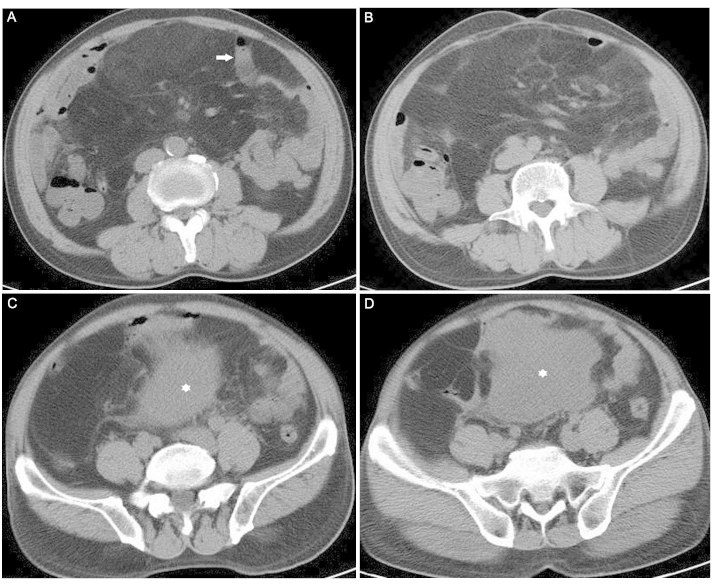
Unenhanced transverse computed tomography images of non-consecutive slices revealing a large, non-encapsulated abdominal-pelvic mass, with predominant fat attenuation. (A and B) The mass demonstrated a heterogeneous texture with coarse septa. A small bowel segment was entrapped in the fatty tissue (A; arrow). (C and D) The tumor also exhibited an inferiorly located area of globular non-adipose tissue, which was partially ill-defined (stars). Small bowel loops were displaced peripherally.

A 59-year-old male was admitted to the emergency department of Yantai Yuhuangding Hospital (Yantai, China) with progressive distension of the abdomen that had been apparent for six months, pyrexia of unknown origin and worsening abdominal pain that had begun five days earlier. A peak body temperature of 38.8°C was recorded, with an associated loss of appetite and fatigue being reported. Blood tests revealed neutrophilic leukocytosis, with an elevated total white blood cell count of 27.94×10^9^/l (normal, 3.5–9.5×10^9^/l), and a differential count of 86.0% neutrophils, which was consistent with infection. Physical examination revealed an ill-defined pelvic-abdominal mass with tenderness in the lower abdomen. A non-contrast abdominal computed tomography (CT) scan performed in the emergency department revealed a large abdomino-pelvic mass, which extended from the upper pole of the kidney to the pelvis. The mass was composed of two components, a main portion of fatty density and a non-adipose solid portion located within the lower part (Fig. 1). The fatty portion was characterized by an extensive infiltrative distribution and the absence of delimitation by a capsule. The mass demonstrated a heterogeneous texture, with coarse septa, patchy soft-tissue components and punctate calcification. The non-adipose solid portion was partially ill-defined and measured 11.6×11.7 cm. The lesion displaced and entrapped the small bowel loops. A pre-operative diagnosis of abdomino-pelvic lipomatosis complicated by infection, was established based upon the clinical characteristics, laboratory results and CT findings.

**Figure 2:**
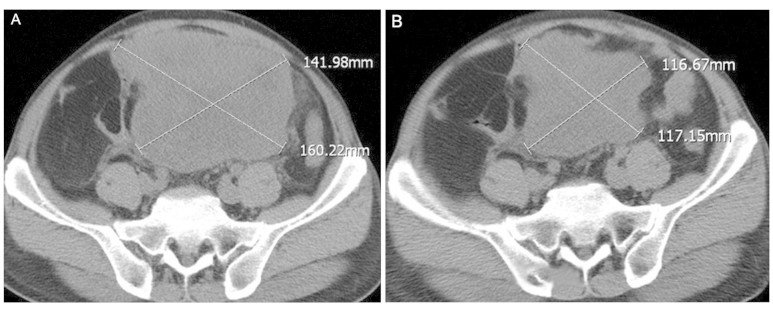
(A) Re-evaluated computed tomography (CT) scan revealing a notable enlargement of the globular, non-adipose tissue eight days subsequent to the inital scan. The tumor measured 14.2×16.0 cm. (B) The initial CT scan revealed that the tumor measured 11.6×11.7 cm.

**Figure 3:**
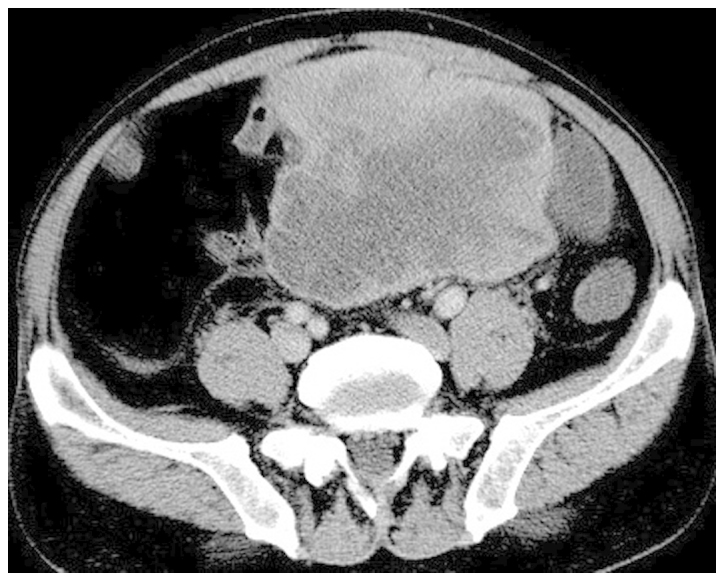
Computed tomography revealing heterogeneous enhancement with hypodense areas in the globular non-adipose tissue.

The patient was admitted to Yantai Yuhuangding Hospital and treated conservatively with broad-spectrum antibiotics. Over the following seven days, the fever remained constant, with temperatures between 37.8 and 38.6°C, and the peripheral white blood cell count remained elevated. On the eighth day post-admission, the mass was re-evaluated using CT intravenous contrast enhancement, which revealed an increase in the diameter of the non-adipose solid mass. The longest diameter measured 14.2×16.0 cm (Fig. 2). The non-adipose solid mass displayed heterogeneous enhancement with hypodense areas, which indicated hemorrhage, necrosis or abscess formation (Fig. 3). The coarse septa and patchy non-adipose components demonstrated minimal homogeneous enhancement.

A laparotomy was performed for symptomatic relief and to obtain a definitive diagnosis. Intra-operatively, numerous separated fatty nodules and masses, with sizes ranging between 1 and 15 cm, were attached to the mesentery of the small intestine. The largest of the masses measured 14×15 cm, was greenish-yellow in color, and was solid with wide areas of necrosis, hemorrhage and abscess formation. The other masses were soft, yellow and homogeneous in consistency. There was no evidence of peritoneal dissemination, ascites or invasion to adjacent organs in the abdominal cavity. The majority of the tumors were separated and removed, but an en bloc resection was impossible to perform due to the large number of small nodular lesions.

**Figure 4:**
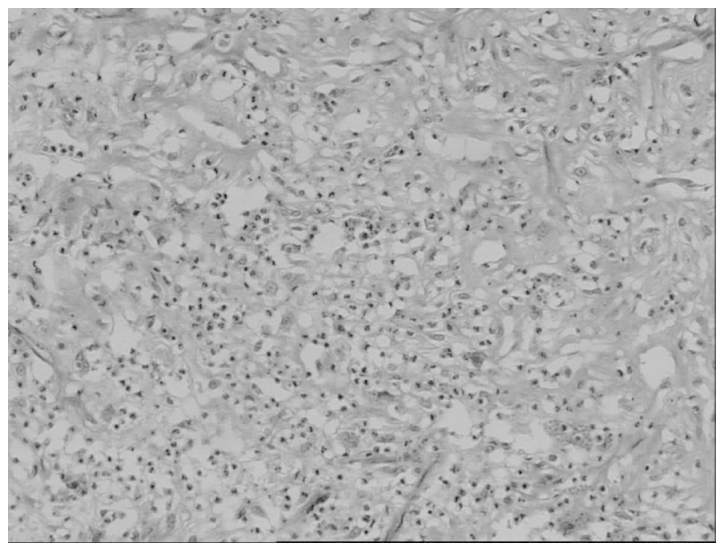
Image of the solid mass revealing a lipomatous tumor with variation in the size of adipocytes. Abundant inflammatory cells, consisting predominantly of neutrophils, were widely distributed throughout the tumor tissue (hematoxylin and eosin staining; magnification, ×40).

**Figure 5:**
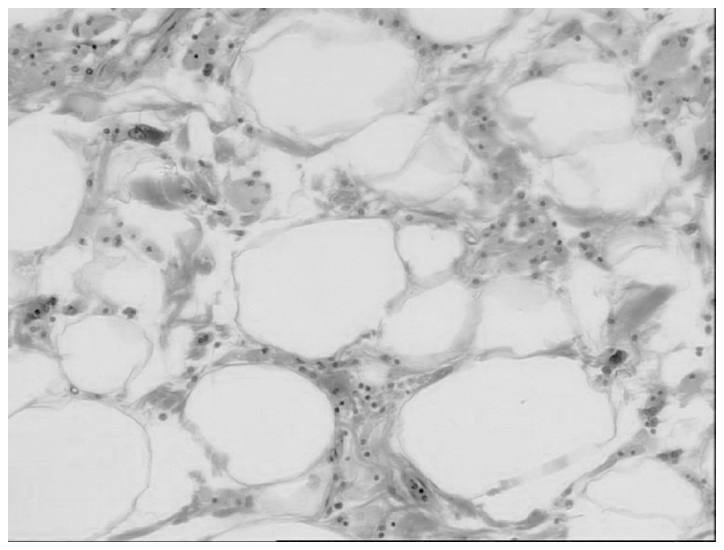
Sections examined from the other nodules and masses demonstrating proliferation of adipocytes, with variations in size and shape. Unusual stromal cells and bistiocytes were scattered within the fibrous septa (hematoxylin and eosin staining; magnification, ×100).

The histological analysis of the solid mass revealed atypical adipocytes with various sizes of fat vacuoles. The abundant inflammatory cells consisted predominantly of neutrophils, which were widely distributed throughout the tumor in the fibrous septa and the adipocytic areas. There were apparent regions of extensive necrosis and hemorrhage, which were densely infiltrated by neutrophils (Fig. 4). The sections from the other nodules and masses demonstrated characteristic histopathological features of a WD liposarcoma, and were composed of atypical adipocytes with various sizes of fat vacuoles. Unusual stromal cells and bistiocytes were scattered within the fibrous septa. Rare lipoblasts were also observed in certain samplings (Fig. 5). Immunohistochemically, tumor cells were positive for S-100 and negative for smooth muscle actin. The final diagnosis was of an adipocytic subtype of multiple mesenteric WD liposarcoma, with the largest of the tumors complicated by purulent inflammation. The patient underwent an uneventful post-operative recovery, and the pyrexia resolved completely following surgery. The patient was discharged on post-operative day 10. No radiotherapy or chemotherapy has been administered in the post-operative period. There were no complaints or complications during the one-year follow-up. A definite abdominal mass was not identified by CT scan performed 12 months after the surgery.

## Discussion

Liposarcoma is a sarcoma of mesenchymal origin, which affects soft tissues of the body, in particular the extremities and the retroperitoneum. On rare occasions, the tumor may affect the mesentery. Primary mesenteric liposarcomas usually arise between 50–70 years of age, and exhibit a higher incidence in males compared with females (3). The clinical presentation can vary, but symptoms often include abdominal pain, distension, the presence of a palpable mass, constipation, vomiting and weight loss (4). In the present study, the patient presented with pyrexia of unknown origin, most likely caused by tumor necrosis and infection.

Histologically, liposarcoma can be divided into five subtypes, myxoid, pleomorphic, dedifferentiated, round cell or WD (5). Upon CT, liposarcoma exhibits contrast enhancement, poor margination, CT attenuation greater than those of normal fat, and inhomogeneity (6). WD liposarcoma can be subdivided into the lipoma-like, sclerosing, inflammatory and spindle cell groups (5). At present, there is no radiologically reliable method that differentiates between these subtypes.Upon imaging, WD liposarcoma appears as a soft-tissue mass, consisting predominantly of adipose cells, with non-lipomatous components. These non-lipomatous features include septa, which are often >2 mm, and small foci, <2 cm in size, consisting of nodular or globular non-adipose tissue (7). In addition, calcifications may be present within the lesion (8). The lesion in the present case was predominantly fatty in nature, with scattered coarse septa, patchy soft-tissue components and punctate calcification, which is consistent with WD liposarcoma.

Multiple mesenteric WD liposarcoma is an extremely rare entity, and at present only one case has been reported in the literature (2). A pre-operative diagnosis of a multiple mesenteric WD liposarcoma may be challenging due to the rarity and lack of awareness of the tumor. The CT features of the lesion in the present study were characterized by an extensive infiltrative distribution of dishomogeneous lipomatous tissue in the abdomino-pelvic cavity, which not only displaced, but also entrapped the small bowel loops. These CT findings differ from those of simple WD liposarcomas, which exhibit well-defined and mainly lobulated margins (1). By contrast, the large non-encapsulated fatty tissue in the abdomino-pelvic cavity in the present case led to a pre-operative diagnosis of lipomatosis, which is a rare benign disease characterized by an overgrowth of non-encapsulated fatty tissue, most commonly in the abdominal and pelvic cavities (9). The large fatty mass of lipomatosis lacks widened septa, and with the exception of its extensive infiltrative distribution, resembles a simple lipoma. In retrospect, the neglect of the multiple coarse septa within the fatty tissue mass, as well as the rarity and lack of awareness of the tumor, are potential reasons as to why multiple WD liposarcoma was not pre-operatively suggested in the present case.

The unusual aspect of the present case was the evidence of necrosis, hemorrhage and abscess formation on a WD liposarcoma. This occurrence appears to be rare, as a PubMed search using the keywords ‘well-differentiated liposarcoma’ and ‘abscess or purulent inflammation’ did not yield any results. The inflammatory tumor in the present study differs from the inflammatory variant of a WD liposarcoma, radiologically and histopathologically. CT revealed that the inflammatory tumor in the present study appeared as a large solid mass with inhomogeneous contrast-enhancement. The imaging features of inflammatory WD liposarcoma are not significantly different from their commoner counterparts (7). Upon microscopic analysis, the inflammatory tumor in the present study was characterized by mature adipocytic proliferation, which was infiltrated by abundant neutrophils. By contrast, the inflammatory cells consist predominantly of plasma cells and lymphocytes in the inflammatory variant of WD liposarcoma (10). Therefore, the inflammatory tumor in the present study does not appear to belong to the spectrum of WD inflammatory liposarcoma.

It should be noted that the presence of large, low-density fat with a large, non-adipose mass may raise the possibility of a dedifferentiated liposarcoma, which is a biphasic WD liposarcoma with a high-grade pleomorphic sarcoma (11). Upon CT, the solid lesions of dedifferentiated liposarcomas are well-demarcated, with distinct planes between fat and solid tumor. The solid mass observed in the present case however was partially ill-defined. Furthermore, in the present case, the re-evaluated CT scan, which was performed eight days after the initial scan, identified a remarkable enlargement of the solid mass. Based upon these CT findings, as well as the presence of leukocytosis and neutrophilia, the most likely diagnosis should be of inflammatory WD liposarcoma, rather than dedifferentiated liposarcoma.

To summarize, the present study reported a case of multiple mesenteric WD liposarcoma, which was complicated by purulent inflammation. The CT features of these multiple tumors were characterized by extensive infiltrative distribution of lipomatous tissue with coarse septa and patchy soft-tissue components. The inflammatory tumor appeared as a large, solid mass and demonstrated heterogeneous enhancement with hypodense areas, which corresponded to hemorrhage, necrosis and abscess formation. Multiple mesenteric WD liposarcoma is an extremely rare entity. This variant should be considered when evaluating a massive infiltration of dishomogeneous lipomatous tissue in the abdominal and pelvic cavity. The present study also revealed that abdomino-pelvic WD liposarcoma can be complicated by purulent inflammation. Radiologists should therefore consider this when establishing differential diagnoses for patients with a fever of unknown origin and the occurrence of a primarily fatty, WD liposarcoma in close proximity to a non-lipomatous mass, as observed in the present study.
